# Acknowledgment to Reviewers of *Micromachines* in 2021

**DOI:** 10.3390/mi13020177

**Published:** 2022-01-25

**Authors:** Micromachines Editorial Office

**Affiliations:** MDPI AG, St. Alban-Anlage 66, 4052 Basel, Switzerland

Rigorous peer-reviews are the basis of high-quality academic publishing. Thanks to the great efforts of our reviewers, *Micromachines* was able to maintain its standards for the high quality of its published papers. Thanks to the contribution of our reviewers, in 2021, the median time to first decision was 14 days and the median time to publication was 32 days. The editors would like to extend their gratitude and recognition to the following reviewers for their precious time and dedication, regardless of whether the papers they reviewed were finally published:
Aabid, AbdulLi, PengAamir, MuhammadLi, PengzhiAbadi, Parisa Pour Shahid SaeedLi, QifeiAbbas, AneesLi, ShanshanAbbas, HaiderLi, ShuangmingAbbas, NaseemLi, SiboAbbasalipour, AminLi, TianAbbasi, AminLi, Tomi T.Abdelhafeez, Ali M.Li, WeijieAbdelkarim, AydiLi, WenmingAbdelmoula, HichemLi, XiangAbdolrazzaghi, MohammadLi, YingsongAbdul Kadir, Aini ZuhraLi, ZhipengAbdullah, Mohammad Faiz LiewLi, ZiliAbdullah, Oday IbraheemLian, GuoxuanAbe, HiroyaLiang, CunmanAbedini-Nassab, RoozbehLiang, KunAbelmann, LeonLiang, PengAboites, VicenteLiao, MeiyongAbreu, CarlosLigęza, PawełAbrol, AmritLillo, Paolo DiAcosta, SerigoLim, An EngAdachi, YoshiakiLim, Chun YeeAdams, Mark L.Lim, Jong-WonAdeela, HanifLim, JormayAfridi, Muhammad IdreesLim, Ki-TaekAgha, ImadLim, SangsoonAghakhani, AmirrezaLima, IvanAgriopoulou, SofiaLin, Chiao-ChiAguas, HugoLin, Chien-hongAhmad, LatifLin, Chih-TingAhmad, Muhammad ShakeelLin, Chih-YuanAhn, Chi BumLin, Chin-FengAhn, Jun KiLin, Chueh-HoAhn, SanghoonLin, Chun-HoAhsan, AMM NazmulLin, Chun-HsiungAinla, AlarLin, Der-YuhAissani, LindaLin, Hung-YinAjami, AliasgharLin, Jia-DeAjejas, FernandoLin, MengjuAjmal, Hafiz Muhammad SalmanLin, Shih-ChiehAkasaka, HirokiLin, Tzu-EnAkkerman, Hylke B.Lin, YangAkkouch, AdilLin, Yu-ShengAkşit, KaanLinardatos, DionysiosAlabrudziński, SławomirLindberg, RyanAlahi, Md Eshrat E.Lio, Giuseppe EmanueleAl-Ameri, TalibLipinski, DariuszAlapan, YunusLipkova, JanaAlazzam, AnasLipovský, PavolAlevras, PanagiotisLitvinova, KarinaAlexandre, AndreaLiu, BoAlexandrovna, Skotnikova MargaritaLiu, BoshenAlgarra González, ManuelLiu, ChangAl-Gburi, Ahmed Jamal AbdullahLiu, ChaoxingAlharbi, MohammedLiu, ChengAlhawari, Adam R.Liu, Cheng-HsienAlho, BrunoLiu, Ching-Ping LiuAli, TanweerLiu, ChuanAliabouzar, MitraLiu, ConghuiAlibakhshikenari, MohammadLiu, GuoAliyu, AliyuLiu, JiaAlkan, BugraLiu, JikaiAlksne, MildaLiu, KunAllahverdi, AbdollahLiu, LiwangAl-Masri, EyhabLiu, LongjuAlmeida, Gustavo Foresto BritoLiu, LumeiAlmubarak, YaraLiu, MingyiAlneamy, AymanLiu, PengfeiAlnis, JanisLiu, Ren-ShiouAl-Sadoon, MohammedLiu, ShulinAlthuwayb, AymanLiu, TairanAltintas, OlcayLiu, TingtingAltynnikov, AndreyLiu, TingyiAlvarez, MarLiu, WenmingAlveringh, DennisLiu, YuxinAly, Mohamed Aly SaadLiu, ZhenyuAmbriz Vargas, FabianLiyakat Hamid, MujawarAmbrożkiewicz, BartłomiejLoi, AngeloAmine, AzizLong, HuAmpeliotis, DimitrisLoo, JackyAmza, CatalinLopes, PaulaAnagnostopoulos, Nikolaos AthanasiosLópez De Lacalle, Luis NorbertoAnanth, AntonyLopez Martinez, Maria JoséAndrade, JoseLopez-Mercado, Cesar A.Andriotis, Eleftherios G.Loschiavo, VincenzoAndrzejczyk, RafałLow, LucieAngiulli, GiovanniLu, MingyangAnguera, JaumeLu, RuochenAngurel, L.A.Lu, TaoAnni, MarcoLucchetta, Daniele EugenioAnoop, GopinathanLucchetta, GiovanniAntonyshyn, IrynaLuchian, IonutAnvar, Amir M.Łuczak, SergiuszAnzagira, LeoLukichev, DmitryAouane, OthmaneLumia, MauroAoyagi, ManabuLunt, Alexander J.G.Aphale, Ashish N.Luo, Guo-LunAral, MustafaLuo, NingqiArash, DaliliLuo, ShaochengAraujo, JeffersonLuo, XiangangAraújo, MárioLuther, Anne-MarieAraújo-Gomes, NunoLuttgen, AndreaAravelli, AparnaLv (Lyu), JianArdakani, Mansoor DashtiLv, ZiyuArdelean, Ioan I.Lynn, Nicholas Scott, Jr.Ardila, GustavoM. Llorens, JoseAretoulaki, EleniMa, ChengfuArith, FaizMa, ChiArsat, RashidahMa, GuangyiArtal, EduardoMa, HanbinArulkumaran, SubramaniamMachno, MagdalenaArvinti, BeatriceMadejczyk, PawelAsano, ShusakuMagnani, AgneseAsgari, SomayyehMagomedov, ArtiomAshraf, NabilMahadik, BhushanAshrafi, NarimanMahajan, GautamAsif, MuhammadMahankali, KiranAšmontas, SteponasMaharbiz, Michel M.Assaf, TareqMaharjan, PukarAstakhov, Viktor P.Mahdavipour, OmidAtaka, ManabuMahmud, Md. AlmostasimAtanasova, PetiaMaile, NageshAtaollahi, NargesMaj, ŁukaszAthanasiou, Christos-EdwardMajee, SubimalAult, Jesse T.Majumder, SutriptoAutrusseau, FlorentMakowski, DariuszAvci, EbubekirMalakoutian, MohamadaliAverbukh, MosheMalankowska, MagdalenaAvis, ChristopheMalboubi, MajidAvram, Marius AndreiMalekimoghadam, RezaAyop, RazmanMalgaretti, PaoloAziz, MuhammadManaf, Asrulnizam AbdBabahosseini, HesamManoufali, MohamedBachman, HunterMantione, DanieleBadea Doni, MihaelaMao, ZebingBadshah, Mohsin AliMarcin Szymon, FilipiakBae, JangPyoMarconcini, PaoloBagolini, AlviseMarcos, MarcosBajić, Jovan S.Marcu, AurelianBakeroot, BenoitMarczewski, JacekBaksa, AttilaMardare, Andrei IonutBalamurugan, RathinamMarek, JurajBalbekin, NikolayMargalit, OdedBaldo, Thaísa AparecidaMargariti, Spiridoula V.Balint, StefanMarian, MaxBallo, AndreaMaric, TijanaBaltatu, Madalina SimonaMarini, MonicaBanan Sadeghian, RaminMarinov, MarinBandyopadhyay, SupriyoMárk, Géza IstvánBanerjee, AishwaryadevMarkovic, TomislavBanerjee, KasturiMarquette, Christophe A.Banerji, SaoniMarsano, DavideBang, JihoonMartin, AndréBansal, KuldeepMartin, IrinaBao, WeizhaiMartinát, StanislavBarabas, RekaMartincic, EmileBarai, Hasi RaniMartínez García, MiguelBarański, AndrzejMartínez López, José IsraelBarbato, MarcoMartínez-Lillo, JoséBarbera, OrazioMartini, ElisabettaBarek, JiriMaruccio, GiuseppeBarettin, DanieleMaruda, Radosław W.Barillaro, GiuseppeMasci, MaurizioBarniol, NúriaMasoero, MarcoBarrino, FedericoMassabuau, FabienBarsan, Madalina M.Mastino, Costantino CarloBarshtein, GregoryMastrangeli, MassimoBartha, KristínaMathew, BobbyBasarab, MikhailMathew, JoiceBasco, LeonardoMatos, Joao NunoBasov, MikhailMatys, MaciejBastmeyer, MartinMauri, RobertoBastos-Arrieta, JulioMauro, Giorgio SebastianoBastounis, EffieMaxime, BavencoffeBasu, PrabalMayer, Alexander E.Basuray, SagnikMaździarz, MarcinBatikh, AhmadMažeika, DaliusBauer, A. J.McCann, RonanBausells, JoanMcCracken, JoselleBautista, OscarMcGrath, DanielBavli, DannyMcNamara, ShamusBayareh, MortezaMd Isa, Illyas BinBayram, AbdullahMebarek Oudina, FatehBeardslee, LukeMei, LanjuBecker, Karl FriedrichMei, LeiBeech, JasonMei, QichangBehbahani, Amir HosseinMei, XutaoBehzad Farahani, BehzadMelin, PedroBeker, LeventMelnikov, AntonBeklemishev, MikhailMendes Junior, José Jair AlvesBelhadi, JamalMendes, MateusBelharet, KarimMeng, DongBella, FedericoMensik, MiroslavBellisario, DeniseMeoni, GabrieleBellutti, PierluigiMercorelli, PaoloBelzunce, Martín AlbertoMerlin, SamBenchea, MarcelinMerlo, SabinaBene, LászlóMerrin, JackBeregoi, MihaelaMeyer, PascalBergamasco, LucaMiao, LudiBerganza Eguiarte, EiderMichael, HeukenBernard, GeffroyMichal, JozwikBerto, MarcelloMichalski, JacekBertolotti, MarioMicheletti, AndreaBertos, Georgios A.Michihata, MasakiBertran, Esteve AmatMidya, RivuBertsch, ArnaudMikołajczyk, TomaszBhadra, NiloyMilani, PaoloBhalla, NikhilMilici, LaurentiuBhatia, DivijMilojevic, AndrijaBhatnagar, ShubhmitaMilovanovic, DubravkaBhattacharya, SaptarshiMin, RuiBhattacharyya, SuvanjanMinea, Alina AdrianaBiagini, GiuseppeMinh, Le VanBichurin, MirzaMiranda, João M.Bień, AndrzejMirea, TeonaBieniek, TomaszMiri, Amir K.Biezma, Maria VictoriaMiricioiu, MariusBigerelle, MaxenceMirza, SabiruddinBijari, AbolfazlMirzajani, HadiBilén, Sven G.Mishra, Geetesh K.Bilican, DogaMishra, NeerajBilski, PiotrMishra, RajeshBin Tamrin, Khairul FikriMishra, RohitBin, YaoMissell, Frank P.Binh, Nguyen Thi ThanhMiszczyk, AndrzejBinsthok, AlexanderMitkowski, PiotrBishop, Greg W.Mitterhuber, LisaBizon, NicuMiyagawa, AkihisaBlack, Bryan JamesMiyauchi, AkihiroBlanco, IgnazioMoayyedian, MehdiBlanton, Eric W.Mobayen, SalehBlažej, JosefMobed-Miremadi, MaryamBloh, JonathanMoccia, MassimoBo, ArixinMoghimi, MohammadBobinger, MarcoMohammad, MostakBocková, MarkétaMohd Najib, Mohd YasinBoćkowski, MichałMohit, MittalBoddaert, XavierMohseni, Parsian K.Bodehou, ModesteMoiseev, KonstantinBoeffel, ChristineMoita, Ana SofiaBoeri, LuciaMojaver, KavehBogdan, GrzegorzMokhtari, FatemehBohdal, LukaszMokrushin, ArtemBolmatov, DimaMoldovan, Carmen AuraBombek, GorazdMoldovanu, SimonaBonaldo, StefanoMolnár, ViktorBonart, HenningMontanaro, AlbertoBorecki, MichalMontemurro, DomenicoBorghi, FrancescaMontessori, AndreaBortone, IlariaMontgomery, Paul C.Bosi, GianniMontillet, AgnèsBothe, Kyle M.Moon, Byeong-UiBou, Santiago FerrandizMoon, ChanwooBourguignon, NataliaMoon, HyejinBoymelgreen, AliciaMoon, InyongBožek, PavolMoon, Joo HyunBozkurt, AyhanMoon, Seung-JaeBozuyuk, UgurMoradi, SaraBrandão, Lincoln CardosoMoradikazerouni, AlirezaBrandl, MartinMorawiec, MateuszBratashov, Daniil N.Morel, AdrienBratsun, DmitryMoretti, GiacomoBrault, JulienMorgan, Joshua TBreazu, CarmenMorikawa, KyojiroBreglio, GiovanniMoritani, KousukeBretterklieber, ThomasMosavati, BabakBretthauer, ChristianMoscatelli, FrancescoBridge, CandiceMoscone, DanilaBronchalo, EnriqueMouli, RamasamyBrouzes, EricMoulianitis, Vassilis C.Bruijn, M.P.Moun, MonikaBruncko, JaroslavMouralova, KaterinaBrunet, PhilippeMouro, JoãoBryja, LeszekMousavi, Mohsen BeladiBrzobohaty, OtoMu, XuanBrzózka, AgnieszkaMukhin, NikolayBu, JianhuiMukhtar, SafyanBudaszewski, DanielMulloni, VivianaBuendia, Carlos DoñateMunos-Camargo, CarolinaBugaj, MarekMünzenrieder, NikoBuj-Corral, IreneMurray, Peter E.Bukatin, Anton S.Murzin, Serguei PetrovichBukosky, ScottMusztyfaga-Staszuk, MałgorzataBunea, Ada-IoanaMuthumariappan, AkilarasanBunge, Christian-AlexanderMuzzio, NicolásBuonamici, FrancescoNa, JongbeomBuoro, Rafael MartosNa, ShuaiBurugupally, Sindhu PreethamNa, TaehuiBurwell, GregoryNabavi, SeyedfakhreddinBusek, MathiasNabavi, Seyedfakhreddin (Koorosh)Butakova, MariaNabil, BelkhirButt, M. AliNadappuram, Binoy PauloseButt, Muhammad AliNadikattu, Rahul ReddyButta, MattiaNagai, MoetoBuzuk, MarijoNaghilou, AidaCabaleiro, ManuelNagit, GheorgheCabot, Joan MarcNagy, GyulaCabrera, HumbertoNajahi Missaoui, WidedCacopardo, LudovicaNajem, JosephCadinoiu, Anca NiculinaNam, InhoCai, JizheNami, MohsenCai, XiaoyangNaphon, PaisarnCaizzone, StefanoNaranjo-Torres, José AntonioCaldas, PauloNaris, SteryiosCaliano, GiosueNasimuddin, N.Caliendo, CinziaNasiri, NoushinCalin, BogdanNasiri, RohollahCalò, GiovannaNassar, AmalCalot, EnriqueNath, SubhasisaCalvo-Lobo, CésarNatu, RuchaCamacho-Leon, SergioNavarro-Cuenca, AnnaCamas Anzueto, Jorge LuisNavickas, RomualdasCametti, CesareNavid, KashaninejadCampanella, HumbertoNavlakha, NupurCampelo, JoséNayak, GauravCampos, Camila D. MadeiraNdieyira, JosephCanet-Ferrer, JosepNejman, MirosławCantero Guisández, José LuisNeumann, NielsCao, ChezhengNeverov, Vladimir N.Cao, Hua-TangNewcomb, RobertCapan, IvanaNezami, SamanCapezza, AntonioNg, Tuck WahCapobianco, EnricoNgo, Ha-DuongCardillo, EmanueleNguyen, Manh-CuongCarelli, GiorgioNguyen, Son ThanhCarou, DiegoNi, QingCarrero, Noelia RubioNiazmand, AmirrezaCarvalho, CarlosNicolaus, MartinCarvalho, Marta S.Niemczewska-Wójcik, MagdalenaCasalino, MaurizioNiemczyk, AgataCasals Terre, JasminaNightingale, AdrianCasañ, GustavoNikishin, SergeyCassinelli, MarcoNikolic, KonstantinCastaldi, GiuseppeNitulescu, GeorgianaCastell, PereNjegovec, MatejCastro-Colin, MiguelNoguchi, YukiCatalan, LeyreNordin, Andrew D.Catalin, DumitrescuNoveanu, SimonaCatarino, SusanaNovitsky, AndreyCavenaghi, MarcosNunna, Bharath BabuCavicchioli, RobertoNurhayati, Retno WahyuCeccarelli, FrancescoObrist, DominikČech, MartinOcchicone, AgostinoCechowicz, RadosławOdry, ÁkosCepeda, JavierOgawa, KensukeCerro-Prada, ElenaOh, TeresaCesano, FedericoOkuducu, Mahmut BurakChae, JoohyungOlaru, DumitruChae, SoosangOletic, DinkoChakrabarti, SomsubhraOliver, C. RyanChamakos, NikolaosOliver, LisaChan, Chin-YiuOliveri, AlbertoChan, Joseph Chang LunOmar, AhmedChandra Adhikari, GopiOmura, YasuhisaChang, Cheng-Hsun-TonyOno, AtsushiChang, Guo-EnOoi, Kim TiowChang, Jen-Yuan (James)Orbulov, ImreChang, Jui-YungOßwald, KaiChang, RuiOstrovidov, SergeChang, Sheng-PoOta, HirokiChang, Yuan-JenOuakad, HassenChang-Liao, Kuei-ShuOuhajji, SamiaChappanda, Karumbaiah NOvchinnikov, MikhailChappel, EricOyunbaatar, Nomin-ErdeneCharles Andrew, DowningOzbayoglu, MuratChaturvedi, MayankOzcelik, AdemChaudhuri, Sandeep K.Ozden, BurcuChekini, MahshidOzerov, MaximChen, ChaohaoOzerova, Anna M.Chen, CheOzkaya-Ahmadov, TevhideChen, ChengpengPacios, MerceChen, Chia-YuanPadhi, Bhaja KrushnaChen, ChihchenPadilla-Medina, Jose AlfredoChen, HaotianPagonis, Dimitrios-NikolaosChen, JiahongPajic, BojanChen, Jian-ZhangPal, JitendraChen, JiayangPal, SnehashisChen, KaikaiPalagummi, Sri VikramChen, KangfuPalestri, PierpaoloChen, MengyuanPalevicius, ArvydasChen, Pang-ShiuPalomares-Caballero, ÁngelChen, Po-WenPaloncýová, MarkétaChen, Shih-HungPan, Ci-LingChen, ShuohanPanas, AndrzejChen, YuchihPanchenko, OlegCheneler, DavidPancrazio, JosephCheng, Chih-HsienPandey, RichaCheng, Ching-HwaPandey, SadanandCheng, Huanyu (Larry)Panetta, DanieleCheng, Ji-YenPanigrahi, BivasCheng, WeirenPanuccio, GabriellaChhetry, AshokPanzardi, EnzaChicos, Lucia-AntonetaPapadakis, Nikolaos M.Chien, Feng-TsoPapadimitriou, Dimitra N.Chirasatitsin, SomyotPapurello, DavideChmielarz, PawełParada Medina, RaúlCho, HanchulPardy, TamasCho, Joon HyongParedes-Madrid, LeonelCho, Kyeong MinParia, DebadritaCho, Seong YunPariente, Cristóbal GarcíaCho, Sung WoonParikh, ChiragCho, SungkwonParinov, Ivan A.Cho, Young-HoPark, Dong HyukChoi, DukhyunPark, HaminChoi, Hae-WoonPark, Je-KyunChoi, HeekyuPark, JihunChoi, HojongPark, JinhyoungChoi, HongsooPark, JisungChoi, JaesoonPark, Jun-YoungChoi, JiyeonPark, Myung HwanChoi, Jong-ryulPark, SeonhwaChoi, Kyeong-KeunPark, Sul KiChoi, Soo HoPark, Sung JeaChoroszucho, AgnieszkaPark, SungjunChowdhury, DhrubajitParmeggiani, MatteoChowdhury, Mohamed FoysolParr, Johnny V VChowdhury, SagarPasadas, FranciscoChung, ChunhuiPascal, JorisChung, JihoonPaschoal, C.W.A.Chung, KunookPashchenko, DmitryChung, Meng-YunPaszkowski, RobertChung, Roy Byung KyuPatel, MalkeshkumarChung, Wen-yaw DannyPatel, SaurinChvála, AlešPaternò, Giuseppe MariaChwastek, KrzysztofPathak, PawanCiarpi, GabrielePatil, Ranjit A.Cimurs, JanisPatra, BishnubrataCiofi, CarminePatterson, Albert E.Ciorap, RaduPaul, SanjoyCiria, MiguelPaulish, Andrey GeorgievichCirillo, GiuseppePauliukaite, RasaCiuffoletti, AugustoPavitra, EluriCiupitu, LiviuPavlin, MaticClaudia, Salanță LianaPavlov, Vitalii A.Coelho, Rodrigo C. V.Payo, IsmaelCofaru, Nicolae-FlorinPedernales, Julen S.Cojocaru, DorianPeixoto De Almeida, MiguelCollins, David J.Pelletier, Mathew G.Colombo, FedericoPeltomaa, RiikkaColombo, Luigi Pietro MariaPeña-Pitarch, EstebanČolović, Mirjana B.Penirschke, AndreasConstantin, Oana EmiliaPerdigones, FranciscoCoppola, GennaroPerec, AndrzejCoppola, SaraPereira Monteiro, PauloCorsaro, CarmeloPereira, SoniaCostanzo, FrancescoPereiro, IagoCosta-Pinto, Ana RitaPerera, NoelCostea, MarianPerfetto, DonatoCoustou, AnthonyPerisin, AnaCovaciu, FlorinPerrin, LaraCovington, JamesPervaiz, SalmanCragg, PeterPesch, Georg R.Crapnell, RobertPetcu, CristianCrescenzi, RoccoPetko, MaciejCrisafulli, GiovanniPetrescu, EmilCristea, Dana MihaelaPetrescu, LucianCristiani, Costanza MariaPetronela, NechitaCristoloveanu, SorinPetrov, MihailCritello, Davide C.Petru, JanaCruz, Juan C.Pezeshki, AtiyeCvek, MartinPfefferkorn, Frank E.Czernohorsky, MaltePham, Duc-ThinhCzerwinska, Areta MagdaPham, HieuCzigany, ZsoltPhan, Thanh LuanDąbrowski, PawełPhani, ArindamDahle, SebastianPierściński, KamilDai, ChangshengPietrowicz, SławomirDai, Qi-linPimenov, DanilDalfré Filho, José GilbertoPin, ChristopheDamej, MohamedPinho, BrunoDamiati, SamarPinho, DianaDaneshazarian, RezaPinna, SergioDanihelová, AnnaPipatpanukul, ChinnawutDanila, OctavianPiper, AndrewDarimireddy, Naresh KumarPires, Ivan MiguelDarvishi, SorourPitas, Charalampos N.Das, AnupamPitchappa, PrakashDas, Gour MohanPivkin, PetrDas, Proloy TaranPiyasena, MenakeDas, SambeetaPizarro, FranciscoDasanna, Anil K.Pizzoferrato, RobertoDasgupta, SayakPlatero, CarlosDashtipour, KiaPlatil, AntoninDasygenis, MinasPlochberger, BirgitDavino, DanielePodwin, AgnieszkaDawwd, ShefaPolese, DavideDayen, Jean FrançoisPolimeni, AntonioDe Cesare, GiampieroPolitano, Grazia GiuseppinaDe Godoy, MarcioPoljak, MirkoDe Lorenzo, GiuseppePollard, Shawn DavidDe Mey, GilbertPolosan, SilviuDe Munari, IlariaPop, NicolaeDe Sabata, AldoPopenda, AndrzejDe Santo, Luca SalvatorePopov, SergeiDe Stefano, LucaPorcu, RobertoDe Vasconcelos, Elder AlpesPortero, AntoniDeaconu, Sorin IoanPortillo-Lara, RobertoDebliquy, MarcPortillo-Vélez, Rogelio De J.Dec, JanPorwik, PiotrDechouniotis, DimitriosPotencki, JerzyDeja, MariuszPotrebic, MilkaDel Giudice, FrancescoPoulopoulos, GiannisDel Hougne, PhilippPoumellec, B.Del Sol Illana, IrenePour, Mohsen SaffariDelattre, CédricPourfattah, FarzadDelgado-Pinar, MartinaPradhan, NiharDelibas, BülentPrakash, M. DurgaDelville, Jean-PierrePrakash, RaviDema, RomanPramanik, TanmoyDen Toonder, J.M.J. (Jaap)Prasad, AlishaDeng, JiePrashant, KumarDeng, PuPremachandra, ChinthakaDeng, SunbinPristavu, GheorgheDens, Jo Andre Y.Prodan, GabrielDerazkola, Hamed AghajaniProux, OlivierDevillard, RaphaelPtaszek, MarcinDevkota, JagannathPuchades, IvanDhakal, RajendraPuna, JaimeDhimish, MahmoudPunetha, AnkitaDi Domenico, GiovanniPurohit, BuddhadevDi Masi, SabrinaPurwidyantri, AgnesDi Schino, AndreaPushpavanam, KarthikDias, Rosana AlvesPustan, MariusDiBella, CalogeroPuszkiel, JuliánDilonardo, ElenaPutz, Ana MariaDing, Ai-XiangPyatnov, MaximDing, HongQasim, MuhammadDinh, ToanQayyum, FaisalDinis, Maria Alzira PimentaQin, KairongDinu Gugoasa, Livia AlexandraQin, ZongDirdal, Christopher AndrewQiu, GangDobre, Robert AlexandruQiu, GuangyuDodero, AndreaQiu, ZhenDodun, OanaQu, ChuangDoi, KentaroQuagliotti, DaniloDolai, SubhashishQuetzeri-Santiago, Miguel A.Doll, TheodorRaad, RaadDolleman, RobinRaatz, AnnikaDominic Lomenzo, PatrickRabbani, GulamDong, KaiRabinovici, RaulDong, LinRabus, DominikDong, XiaoguangRadadia, Adarsh D.Dong, ZiyeRadek, NorbertDonkers, Joanne M.Radner, HannesD’Orazio, FrancoRadočaj, DorijanDorenbos, GertRadu, Ionut-CristianDorp, Dennis VanRady, MohamedDoutch, JamesRaeis-Hosseini, NiloufarDrago, CarloRafiq, Md Khan Sobayel BinDriesche, Sander Van DenRahaman, AshiqurDriscoll, NicoletteRahman, MizanoorDu, E (Sarah)Rahman, MuhibUrDu, HejunRahman, Muhommad AzizurDu, MingdeRahmani Ahranjani, RaminDuca, LuciaRahmani, RahimDudek, MichalRahmani, RaminDueñas Carazo, SalvadorRaj, Nirmal Prashanth Maria JosephDun, ChaochaoRajappan, AnoopĐurek, IvanRajesh, A.Dussoni, SimeoneRajput, ShailendraDuttaGupta, SamikRamakrishnan, N.Duvigneau, FabianRamer, GeorgDuy, Le ThaiRamón-Azcón, JavierDyl, TomaszRamos, DanielDzedzickis, AndriusRamos-Organillo, ÁngelDzionk, StefanRana, Abu Ul Hassan SarwarDziurzanski, PiotrRana, PuneetEbada, Ahmed IsmailRanitović, AleksandraEbbens, StephenRapisarda, MatteoEdwardraja, SelvakumarRashid, Rizwan RahmanEfthymiadis, PanosRashidi, Mohammad MehdiEfthymiou, LoizosRastin, HadiEglitis, RobertsRatzke, MarkusEhrmann, AndreaRaza, AliEichberger, BerndReddy, N. SubbaEielsen, Arnfinn AasReddy, Y. Veera ManoharaElafandy, RamiRees, D. Andrew S.Elbuken, CaglarRego, GasparElgeneidy, KhaledRehman, Muhammad MuqeetEllahi, RahmatReig, CàndidEllendt, NilsReis, JoãoElumalai, PalaniRella, RobertoEmadi, ArezooRemes, ZdenekEminoglu, BurakRen, DingkunEom, KyungsikRen, GuanghuiErten, Ahmet CanRen, HaoEscobedo, CarlosRen, JifengEsmaiel, HamadaRen, Shun-qingEsposito, FlavioResetic, AndrazEstevadeordal, JordiReuben, JohnEvans, BenjaminReuter, DannyEwart, Paul D.Reuter, FabianEzquerra, TiberioReyes-Belmonte, Miguel A.Faccio, GretaRezvani, MohsenFagadar-Cosma, EugeniaRhee, HuinamFal, JacekRho, Hoon SukFalconi, ChristianRibeiro Junior, Luiz AntonioFan, Ching-LinRibeiro, JoãoFan, Yu-JuiRibeiro, Paulo A.Fang, BaizengRicci, StefanoFang, ChengchengRichards, Dylan JackFang, FengzhouRidhar, Varun SFang, GuozhaoRigo, RiccardoFang, RuixianRimkus, AlfredasFara, LaurentiuRinklin, PhilippFarago, PaulRipka, PavelFarahani, Behzad V.Rizal, ConradFarajpour, AliRizzello, GianlucaFarhangdoust, SamanRobert, NewcombFarrugia, RussellRoberts, Robert C.Favalli, MicheleRobichaud, AlexandreFeeney, AndrewRocco, DavideFelba, JanRoch, Alexandre LeFeng, HaidongRocha, Rodrigo TumolinFeng, ShilunRodkevich, NikolayFeng, ZhihuaRodrigues, Mário S.Fernandes, ElisabeteRodrigues, Tiago A.Fernandes, F.Rodríguez-Reséndiz, JuvenalFernandez, RoemiRohani, Mohamad Nur Khairul HafiziFerrante, CarinoRohner, PatrikFerraro, DavideRojas-Cárdenas, MarcosFerri, ElenaRolan, AlejandroFerri, James K.Romanov, AleksandrFiala, PavelRosa Rodriguez, MontañesFiáth, RichárdRosa, Luis GuerraFicial, BenjamimRosenberger, A.T.Fick, JochenRoshanghias, AliFidan, IsmailRosi, AntonellaFilip, PetrRossella, FrancescoFilipič, BratkoRothemund, PhilippFilippov, AndreyRovisco, AnaFirebaugh, SamaraRowe, BrianFischer, GeorgRoy, BibhasFitzek, HaraldRoy, TamalFobelets, KristelRoyson Donate, DsouzaFolch, AlbertRóżowicz, AntoniForesti, RubenRuan, HaihuiFornell, AnnaRubio, Jorge MorenoForouzandeh, FarzadRubtsov, NikolayFournel, FrankRucki, MiroslawFowler, ClaytonRudakova, Natalya V.Frackowiak, StanisławRudnicki, KonradFrança, Thiago ValleRuggeri, SerenaFrascio, MattiaRumbo Morales, Jesse YoeFreudenberger, JürgenRumyantsev, SergeyFroemel, JoergRupcic, SlavkoFrutos, SergioRusu, CristinaFu, DongRuszaj, AdamFu, LiRybarczyk, Maria K.Fu, Lung-MingRyuzaki, SouFujii, SatoshiRyynänen, TomiFujimoto, DaisukeSaadatmand, MaryamFujinami, KaoriSaccon, LeonardoFukushima, TakafumiSadabadi, HamidFunazuka, TatsuyaSadati, MonirosadatFutai, NobuyukiSadeghi Bigham, BahramFydrych, DariuszSadowski, ŁukaszGaddam, AnveshSaeed, AnwarGagniuc, PaulSaeidi, RoghayehGajic, DragoljubŠafarič, RikoGallaher, JillSaffell, JohnGalletti, ChiaraSaha, ChittaGamella Carballo, MariaSaha, RenataGangadharan, RajanSahoo, SumantaGanguly, SamiranSahoo, SurjitGangwar, KapilSahu, ManishaGarcia Arribas, AlfredoSaid, MuzalifahGarcía-Barriocanal, JavierSaija, RosalbaGarcia-Lechuga, MarioSakač, NikolaGarmendia, IñakiSakai, TakeyasuGarten, LaurenSakellis, EliasGas, KatarzynaSałaciński, TedeuszGas, PiotrSalahdine, FatimaGasparski, AlexanderSalapare, Hernando IIIGassmann, StefanSalari, AlinaghiGatty, Hithesh KumarSalauddin, MdGaudiuso, CaterinaSaleem, ArslanGazda, PiotrSaleem, Muhammad MubasherGe, WenjunSalerno, NunzioGębara, PiotrSalman Al Farisi, MuhammadGedvilas, MindaugasSalman, MootazGeethanjali, M.Salzenstein, FabienGennari, ClaudioSalzenstein, PatriceGeonea, Ionut DanielSamal, SnehaGeorgiev, DejanSamaras, IoannisGeorgiou, Emmanuel P.Sambasivam, SangarajuGepp, Michael M.Samiei, EhsanGerasimov, JenniferSampaio, PaulaGerber, DoronSancataldo, GiuseppeGergidis, Leonidas N.Sanchez-Rojas, Jose LuisGerhardt, NilsSanctis, ShawnGervaso, FrancescaSandeep, Chitta SaiGesuele, FeliceSandner, ThiloGeszke-Moritz, MałgorzataSanseverino, AnnunziataGevorkov, LevonSantangelo, Paolo E.Ghani, Ihsan AliSantos, JerranGhasemi, RastaSarabia-Vallejos, Mauricio A.Gheorghiu, MihaelaSaremi, MehdiGhorbani, FarnazSarikov, AndreyGhorbani, MortezaSarkar, AninditaGhosh, AbhishekSarker, SubrataGhosh, SoumenSarris, Ioannis E.Giacomozzi, FlavioSasaki, NobuoGiannatsis, JohnSathish, ShivaniGiaquinto, MartinoSato, RyutaGibson, JamesSavu, Sorin VasileGil Santos, EduardoSawabata, NoriyoshiGilbert, Hunter B.Sayedi, Sayed MasoudGill, ElisabethScaffardi, M.Ginnaram, SreekanthSchaefer, Hans-EckhardtGisbert-Garzarán, MiguelSchill, GregoryGismalla, Mohammed Salih MohammedSchill, MatthewGkrintzalis, KonstantinosSchimpe, MichaelGoda, KeisukeSchirhagl, RomanaGodec, DamirSchliwa, AndreiGogolewski, DamianSchmidt, GeorgGöktaş, HasanSchnaubelt, SebastianGołąbczak, MarcinSchnorr, JörgGolgovici, FlorentinaScholl, Philipp M.Golijanek-Jędrzejczyk, AnnaSchroeder, GrzegorzGomes, Henrique LeonelScognamillo, CiroGomez, AnaSecciani, NicolaGonçalves, Helena M. R.Secuianu, CatincaGonchar, KirillSedlák, PetrGong, ChaoyangSee, Tian LongGong, MaxSegura-Cárdenas, EmmanuelGonzalez-Ayala, JulianSeichepine, FlorentGonzález-Garcinuño, ÁlvaroSeidel, KonradGopu, Sriram GopuSemenov, MikhailGórecki, PawełSenatov, Fedor S.Gorjanc, Dunja ŠajnSeo, GiwanGoswami, DebkalpaSeo, HwajeongGrabowski, MiroslawSeo, JongbumGraceffa, RitaSeo, SehunGraczykowski, BartlomiejSeok, OgyunGraf, MarkusSeok, SenhoGrangeat, PierreSergi, ConsolatoGrasman, JonathanSergides, MariosGregušová, DagmarSerhatlioglu, MuratGrill, RomanSerra, EnricoGrisé, FabienSerra, MarcoGroenewold, JanSerrano, Javier ToledoGrosjean, GalienSerrano, Jose JavierGrossi, MarcoSerry, MohamedGrumezescu, Alexandru MihaiSgroi, Mauro FrancescoGrunwald, GrzegorzShaburova, NataliyaGrunwald, RuedigerShadloo, Mostafa S.Grunwald, TimShahsavan, HamedGrzybek, DariuszShaikh, RubinaGuan, ZeyiShajari, ShaghayeghGuariglia, EmanuelShaker, AhmedGueorguiev, Gueorgui KostovShakoor, AdnanGui, LinShama, FarzinGui, XuchunShamanaeva, Liudmila G.Guidi, FrancescoShamonin, MikhailGuitián, FranciscoShang, LuoranGulari, ErdoganShankar, BhawaniGulbeyaz, OnderShankles, PeterGündoğan, MustafaSharafeldin, MohamedGuo, FengShardt, NadiaGuo, JiminSharker, Shazid Md.Guo, JingshuSharma, AnuragGuo, WeijinSharma, Piyush SindhuGuo, XiaoguangSharma, RamakantGupta, MunishSharma, RichaGupta, RohitShavezipur, Mohammad K.Gupta, SanjuShcherbakov, AlexanderGu-Stoppel, ShanshanSheard, SteveGutiérrez Ramírez, ManuelShemelya, CoreyGutruf, PhilippShen, ChenGuzmán, EduardoShen, HaotingGyawali, GobindaSheremet, MikhailHa, Ngoc SanSheridan, Richard JHagelauer, AmelieShevchenko, Vitaliy G.Hahm, Sung HoShevchik, Sergey A.Hai, ShafiqulShi, LeileiHaidegger, TamásShi, QiongfengHajjaj, Amal Z.Shi, ShengfangHalder, RaktimShigemune, HirokiHamad, EyadShikler, RafiHamza, RafikShiku, HitoshiHan, DongdongShimizu, JunHan, Gyeo-ReShin, JoonghanHan, JianingShin, MinchulHan, MengdiShin, SangwooHan, WeinaShinde, Surendra KrushnaHanasoge, SrinivasShinshi, TadahikoHannu, JariShirota, MinoriHao, NanjingShittu, SamsonHappy, HenriShoffstall, AndrewHaq, Ijaz UlShoji, KanHaque, A. B. M. TahidulShrirao, Anil B.Haque, Razi-ulShukla, AtulHara, MotoakiSiami-Namini, SimaHarding, BrendanSicard-Roselli, CécileHarea, EvgheniiSiddiqui, Fahad ManzoorHartnagel, HansSiebenhofer, Matthäus S.Hasan, Md SakibSieber, IngoHasanzadeh Kafshgari, MortezaSienkiewicz, ŁukaszHashemi, Seyyed AlirezaSignore, GiovanniHassanien, AbdouSilaghi, Andrei MariusHasse, SybilleSiller, HectorHatamie, AmirSilva, Joao FranciscoHavran, LudekSimanjuntak, Firman MangasaHe, TianyiyiSimic, MarkoHébrard, LucSimić, MitarHedhammar, MySimon, BertrandHejazian, MajidSimorangkir, Roy B. V. B.Helerea, ElenaSimoska, OljaHemsel, TobiasSimo-Tagne, MerlinHernandez-Machado, AuroraSing, Swee LeongHernandez-Matamoros, AndresSingh, Ajay VikramHernando-Garcia, JorgeSingh, MadhavHerrera-May, Agustín LeobardoSingh, Narendra K.Herth, EtienneSingh, PragyaHesketh, PeterSingh, RiturajHess-Dunning, AllisonSingh, Sajal SagarHeymann, MichaelSinko, PatrickHildebrand, JoergŠipoš, MartinHillman, RobertSiragusa, SergioHippler, RainerSirenko, AndreiHirai, YoshikazuSirico, DomenicoHiraishi, TetsuyaSitar, AnžeHjelmervik, Jon MikkelsenSivarupan, TharmalingamHlawitschka, MarkSivasubramanian, JayaharHlosta, JakubSkliros, DimitriosHoffman, JacekSkonieczny, KrzysztofHoffmann, AndreasSlatineanu, LaurentiuHofstetter, DanielSleczkowski, PiotrHohmann, TimŚliwa, IzabelaHolloway, PaulŚliwiński, PawełHong, AyoungŚlusarczyk, ŁukaszHong, Chang-KiSmagin, NikolayHong, Chih ChiangSmart, Luke R.Hong, Ic PyoSmith, StephanieHong, SeongjinSnopiński, PrzemysławHopper, RichardSo, ByeongchanHošek, JanSoares Dos Santos, Marco P.Hosseini, Seyed AliSobotka, PiotrHou, ZhilingSoda, JoskoHsiao, Hui-HuaSoetedjo, AryuantoHsieh, Chien-WenSoh, Jun HuiHsieh, Chih-ChunSohel, Shahadat HasanHsu, Chai-HsienSojahrood, Amin JafariHsu, Heng-TungSokolov, SergeyHu, ChengliangSokolova, Maria PetrovnaHu, GuobiaoSoler, Miguel A.Hu, JieSołoducho, JadwigaHu, JunSolodukhin, Alexander E.Hu, Kai-MingSomlyai, GáborHu, LeiqingSonar, SantoshHu, LiangxingSong, ‪HengxuHu, SiyiSong, Hyun-CheolHu, Yi-LiangSong, Seung-HwanHua, QilinSong, ShuangHuang, CanSong, YangHuang, Cheng-ShengSong, Yong-Ak (Rafael)Huang, Chia-YenSong, YuanpingHuang, Daniel ZhengyuSonnino, GiorgioHuang, HanSornin, DenisHuang, Jen-HuangSorrell, J. MichaelHuang, Ming-ShyanSosa González, Carlos JavierHuang, Po-TsangSosnowski, MarcinHuang, QijinSotiriadis, PaulHuang, QinglanSoto Rodriguez, Paul Eduardo DavidHuang, QiuliangSoto, FernandoHuang, ShuiquanSoto, RobertHuang, WeiminSouayeh, BasmaHuang, XudongSoucemarianadin, ArthurHui, Teo TeeSoucy, Jonathan RHuignard, Jean-PierreSowinski, PrzemyslawHuling, JenniferSpeidel, AlistairHumayun, MuhammadSpièce, JeanHung, Shao-KangSpina, RobertoHuo, SuguoSplinter, RobertHuotari, MattiSreedharan, SreejeshHuq, SaifSreenivasan, Sreeprasad T.Hur, ShinSridhar, AdithyaHussain, Khalil KhadimSridharan, AratiHussain, NiamatSrinivasa Rao, PedapatiHutchens, Thomas C.Sriram, RashmiHuynh, Thanh ThuongSrivastava, AnubhavHuynh, Thanh-CanhStachiv, IvoHvizdoš, PavolStachowicz, FeliksHwang, ChangmoStahl, FrankHwang, David J.Stamatelos, TassosHwang, Gyu WeonStanchu, HryhoriiHwang, Jun YoungStanley, JohnHwang, Yin-TsungStarace, GiuseppeHwu, Edwin En-TeStarman, LaVernHwu, Jenn-GwoStarosta, RobertHyer, HoldenStassi, StefanoI. Hernández, MartaStaszuk, MarcinIannazzo, DanielaStavrakis, StavrosIgreja, JoséSteyer, DanielIhmig, Frank R.Sthal, FabriceIkehashi, TamioStieglitz, ThomasIkerionwu, CharlesStiharu, IonIliescu, CiprianStolarczyk, AgnieszkaIliescu, MihaielaStortini, AngelaIllés, BalázsStraka, L’uboslavIlyushin, Yaroslaw A.Strauss, IvoIno, KosukeStrozzi, MatteoInomata, KatsuhiroStrušnik, DušanIoana, AdrianStrzałkowski, KarolIoannidou, Melina P.Subramanian, BalajikarthickIoannis, TheodorakosSuciu, GeorgeIon, MarianSuganthi, S. T.Ionete, Eusebiu IlarianSuherman, Alex L.Iqbal, FaisalSujecki, SlawomirIqbal, FaizSukumaran, AnilIrshad, KashifSułkowicz, PawełIshida, TadashiŠumak, BoštjanIshida, YuichiSun, BaiIslam, Md. MydulSun, DaliIslam, Md NazmulSun, DiIslam, Md RabiulSun, EnweiIslam, MonsurSun, GongchenIslam, ZahabulSun, HongbinIsozaki, AkihiroSun, Kien-WenIto, ManabuSun, MengIwami, KentaroSun, XinIzawa, Kazuhiro P.Sung, JaeyongIzquierdo, AlbertoSung, Young JoonIzzetti, RossanaSunil, PathakJackson, MarkSunny, TomJackson, Mark JamesSurappa, SushrutaJackson, NathanSurdo, SalvatoreJadwicienczak, WojciechSusarrey-Arce, ArturoJahanbin, AminhosseinSušić, AleksandarJahangir, IfatSuzuki, DaisukeJakła, SlawomirSuzuki, MasashiJakubowska, BlankaSuzuki, MasatoJalili Firoozinezhad, SasanSuzurikawa, JunJamalabadi, Mohammad Yaghoub AbdollahzadehŚwiercz, RafałJanas, DawidSymons, DigbyJang, BumjinSypniewska-Kaminska, GrażynaJang, JaewonSzachogluchowicz, IreneuszJanissen, RichardSzarek, ArkadiuszJankowski-Mihułowicz, PiotrSzczech, MarcinJanos, RudolfSzermer, MichalJanusas, GiedriusSzewczyk, RomanJarwar, Muhammad AslamSzielasko, KlausJáuregui-Vázquez, DanielSzkulmowski, MaciejJaved, SofiaSzulczyński, BartoszJayababu, NagabandiSzwajca, AnnaJefimovs, KonstantinsTabassum, ShawanaJen, Chun-PingTaboryski, Rafael J.Jenkins, DavidTada, ShigeruJeon, JongwookTagliavini, GiuseppeJeon, SunghoTaherdangkoo, RezaJeong, Ok ChanTai, Li-ChiaJeong, SunghoTait, NiallJerzemowska, GrażynaTakagi, ShuJhabvala, JamaspTakei, HiroyukiJi, JianlongTakeishi, NaokiJian, ShunyiTalanov, Mikhail VJiang, ChanghuiTalebi, Sayed PouriaJiang, FengTaler, JanJiang, JiekeTamarin, OllivierJiang, LaimingTamersit, KhalilJiang, MengTamus, Zoltán ÁdámJiang, TaoTan, ChristabelJiang, Tsin-FuTanabe, MasayukiJiannan, LiTanaka, NobuyukiJilani, Syeda FizzahTandecka, KatarzynaJiménez-Barbero, JesusTang, ShiJiménez-Molinos, FranciscoTang, Shi-YangJiménez-Pérez, RebecaTangirala, Venkata Krishna KarthikJin, CongruiTani, HirofumiJin, QinghuiTapajna, MilanJinoop, A.N.Tarasov, Anton S.Jnawali, KamalTatar, ErdincJo, JanggunTayebi, TaharJo, Jeong-WanTeixeira, EduardoJohannesson, DanielTeixeira, Marcos F.S.John, Rohit AbrahamTelada, SouichiJohnson, BenTéllez-Limón, RicardoJonda, EwaTeo, JeremyJones, LeeTéot, LucJoshi, NiravTeshima, Tetsuhiko F.Joshi-Imre, AlexandraTessore, FrancescaJourlin, MichelTestani, ClaudioJovičević-Klug, MaticThakur, RavirajJovicevic-Klug, PatriciaThangam, RamarJuárez, Jaime JavierThein, Chung KetJudit, TelegdiThiery, LaurentJugessur, AjuThom, ChristianJung, Jae HwanThomas, NoelJuodkazis, SauliusTian, Fang-BaoJurca, FlorinTiberiu Petrescu, Florian IonK. Imlau, MircoTiggelaar, Roald M.Kacem, NajibTikadar, AmitavKafle, BishalTiliakos, AthanasiosKage, AzusaTipeev, AzatKahng, SungtekTkáč, JánKaiser, ThomasTkach, ItshakKajita, MasatoshiTobet, StuartKakoti, AnkanaTodorov, RossenKamat, Amar M.Tognarelli, SeleneKamebuchi, HajimeToledo, JavierKamedulski, PiotrTomasiunas, RolandasKamei, Ken-ichiroTomaszewski, DanielKampa, AdrianTomov, Martin L.Kämpfer-Homsy, AlexandraTonelli, Alan EdwardKanclerz, PiotrToney, James E.Kang, ChanKyuToroń, BartłomiejKang, HyeminTorregrosa, AdrianKang, MinATorres-Mendieta, R.Kang, Seung-KyunTorres-Sospedra, JoaquínKang, XuanwuTorzewski, JanuszKaniowski, RobertToshiyoshi, HiroshiKanjilal, BaishaliTowe, BruceKant, KrishnaTran, Duy ThanhKao, Kuo-ShengTrigili, EmilioKaplar, RobertTrinh, Quang-KienKapsalis, Vasilios C.Tripathi, NishantKarabchevsky, AlinaTristo, GianlucaKaradimitriou, NikolaosTrpčevska, JarmilaKarakasidis, TheodorosTrujillo, CarlosKaramanos, Theodosios D.Trusiak, MaciejKarczemska, AnnaTryznowski, MariuszKärnfelt, CamillaTsai, Chin-ChunKarsai, ArpadTsai, Paul Hsieh-FuKarumuri, Srinivasa RaoTsakalis, KonstantinosKarvelas, EvangelosTsao, Chia-WenKasendra, MagdalenaTsao, Chung-ChenKastyl, JaroslavTselev, AlexanderKathuria, HimanshuTseng, Sheng-HsiangKatsikis, GeorgiosTsongas, KonstantinosKatzschmann, Robert K.Tsukagoshi, HideyukiKaur, HarsupreetTsutsui, MakusuKazanskiy, NikolayTuohiniemi, MikkoKazazis, DimitrisTuralchuk, PavelKeating, AdrianTwu, Ruey-ChingKecskes, IstvanTyaginov, StanislavKelemen, MichalUeno, KoheiKemmegne-Mbouguen, Justin ClaudeUherek, FrantisekKenry, KenryUllah, RehmatKeresztes, ZsófiaUngurean, IoanKeskinbora, KahramanUnterreiner, Andreas-NeilKhachaturian, AroutinUrbanowicz, MarcinKhaliel, MaherUrbanowicz, TomaszKhalil, IslamUrbikain Pelayo, GorkaKhamas, SalamUreshino, HiroshiKhan, AsifUshida, AkiomiKhan, FazlurrahmanUtsuzawa, ShinKhan, IftekharUvarov, Ilia V.Khan, Mohammad Nasim ImtiazV. Povolotskiy, AlexeyKhan, MuhammadVadavalli, SaikiranKhan, RajuVahidi, Nasim WinchesterKhan, Sabbir A.Vannier, MichaelKhan, SovannVardasca, RicardoKhandelwal, GauravVarga, DomonkosKhani, ShaghayeghVarga, GáborKhezri, BaharehVarga, PalKhodayari Bavil, AliVargas Salgado, Carlos AfranioKhope, Akhilesh S. P.Vargas-Bernal, RafaelKiani, AmirkianooshVargas-Chable, PedroKiazadeh, AsalVarin, Robert A.Kiiski, IiroVarum, TiagoKikionis, StefanosVasile, AlexandruKikuchi, HiroakiVasilev, SemenKillgore, JasonVasilyeva, MariaKillian, ManuelaVazquez-Avila, Jose LuisKim, BongjoongVediappan, Rajan S.Kim, Byeong HeeVega, Gisela RuizKim, Choong HyunVeiga, Manuel FernándezKim, Chul MinVela, Emir A.Kim, Dong MinVelazquez-Perez, Jesus E.Kim, Dong-SeokVelea, Marian NicolaeKim, GaramVelez Cuervo, CamiloKim, GunzungVendik, Irina B.Kim, Hae-JinVenzac, BastienKim, Heuy DongVercelli, BarbaraKim, HowukVerd, JaumeKim, Hwan D.Verona, EnricoKim, HyoncholVeronesi, FedericoKim, InkiVersaci, MarioKim, JaemyungVetturi, DavidKim, JeonghyunVihar, BoštjanKim, Jin YoungVila-Comamala, JoanKim, JinsikVillarejo Mayor, John JairoKim, Jong MinVillone, Massimiliano MariaKim, JoohanVisaveliya, NikunjkumarKim, Jung-MuVita, AlessioKim, JungsikVittori Antisari, MarcoKim, JungwooVizureanu, PetricaKim, KeekyoungVladimir, KodnyankoKim, Ki SeokVlcak, PetrKim, Ki-IlVoicu, RodicaKim, KwangeunVolegov, Aleksey SergeevichKim, KwangsooVollbrecht, JoachimKim, Min-SuVolpe, AnnalisaKim, Moon DeockVossou, ClioKim, Moon-SeokVrabec, TinaKim, Seon JoonVrsaljko, DomagojKim, SoheeVukoje, MarinaKim, Sung JaeWahaia, FaustinoKim, Sung SooWang, AndongKim, SungjunWang, AnyangKim, SunmeanWang, BaomingKim, TaeyoonWang, BinKincses, AndrásWang, BinghaoKing, Justin B.Wang, BoKintzios, SpyridonWang, CemingKirchhoff, MichaelWang, ChaoKiss, ImreWang, ChaofuKitahama, YasutakaWang, ChenKitano, ClaudioWang, Chien PingKlačková, IvanaWang, Chih-LiangKlepacki, DariuszWang, ChunjinKlimley, Abbott PeterWang, Dung-AnKłonica, MariuszWang, GeKlos, Jaroslaw W.Wang, HaiyangKloskowski, TomaszWang, JianKluczynski, JanuszWang, JieKmita, AngelikaWang, JinliKnápek, AlexandrWang, Jung-ChangKnapkiewicz, PawelWang, KaiyouKnapp, Gerald L.Wang, KangKnypiński, ŁukaszWang, LeiKo, JinaWang, LingKobayashi, MakikoWang, LiuKobtsev, SergeyWang, MohanKocik, MarekWang, Mu-chunKodet, JanWang, QinghuaKoh, DominWang, Shau-ChunKoh, Kah HowWang, ShiyanKoichiro, NambuWang, ShueKokkoris, GeorgeWang, Sying-JyanKolesov, VladimirWang, TaoKolhatkar, GitanjaliWasige, EdwardKomada, PawelWasilewski, TomaszKombaiah, BoopathyWeber, MatthieuKondinski, AleksandarWegiel, TomaszKong, Choon YenWei, JingxuanKong, JoonhoWei, TiweiKontoni, Denise-PenelopeWeinberger, ChristianKoo, ChiwanWeißmantel, SteffenKoolivand, AbdollahWeiwei, ShiKoral, CanWen, HaoranKoreanschi, AndreeaWen, Ji BoKorganbayev, SanzharWendisch, Fedja J.Korobiichuk, IgorWendt, ThomasKoronkiewicz, StanisławaWenzhong, LouKorsunsky, Alexander M.Westerhausen, ChristophKorytar, DusanWhite, Ryan T.Kose, TalhaWisniewski, PiotrKosmidis, LeonidasWitkowska Nery, EmiliaKost, Gerald J.Wittemann, AlexanderKoszewnik, AndrzejWittemann, FlorianKotlyar, Margarita V.Woafo, PaulKouh, TaejoonWojciechowski, SzymonKouziokas, Georgios N.Wójcik, DariuszKowalski, BogdanWojnicki, MarekKozochkin, Mikhail P.Wong, BryanKramberger, ChristianWong, Kin ShunKrasuski, KamilWong, Pak KinKrawczyk, JacekWoo, JiyongKrebsz, MelindaWoo, KyooheeKremieniewski, MarcinWoo, Seong TakKrenicky, TiborWośko, MateuszKručaitė, GintarėWozny, JanuszKrueger, EddyWrobel, AndrzejKrüger, BjörnWróblewski, RafałKrukiewicz, KatarzynaWu, Chao-HsinKrumpolec, RichardWu, Chia-CheKryzhanovskaya, NataliaWu, Chih YangKubasov, IlyaWu, Chyan-ChyiKuczmaszewski, JozefWu, GuangfuKudr, JiriWu, HuijuanKühne, ThomasWu, LixiangKukaev, AlexanderWu, Shin-TsonKukhtevich, IgorWünsche, ChristineKukushkin, VladimirXavier, MiguelKula, SławomirXiang, JiwenKulinich, SergeiXiao, JunjunKulsharova, GulsimXiao, MingKumar Thakur, MukeshXie, HuikaiKumar, AkshayXiong, BinKumar, AnujXiong, HaoKumar, ArvindXu, JinzeKumar, AvneeshXu, JiushuaiKumar, ManishXu, MinKumar, Mukesh, Dr.Xu, Nicole W.Kumar, PunitXu, RuoyuKumar, RajeshXue, FenKumar, SachinXue, HaoranKumar, ShailabhYahya, Salah I.Kumari, PreetiYamada, AyakoKumchai, HattanasYan, JiaqiKuncser, Andrei CristianYan, ShengKung, Chung-WeiYang, Chih-ChinKuntsevich, Alexander YuYang, FeipengKunwar, PuskalYang, HailinKurc, BeataYang, JunjieKurita, KazunariYang, ShujieKurokawa, SyuheiYao, Da-JengKusetogullari, HuseyinYapici, Murat KayaKusior, AnnaYasa, Immihan CerenKutschmann, PiaYasuhiro, MiyasakaKuz’min, Mikhail P.Ye, FanKuznetsova, YuliaYe, HangKwak, Tae JoonYeh, Li-HsienKwon, Oh-KyongYeo, Joo ChuanLabardi, MassimilianoYi, HwangLabun, JánYih, Chih-ChenLacerda, RobertoYildirim Taser, PelinLachowicz, MarzenaYildiz, FikretLackowski, MarcinYilmaz, OguzhanLafata, PavelYin, HeyuLaguarda-Miró, NicolásYing, BinbinLai, MeimeiYioultsis, Traianos V.Lai, StefanoYoo, HocheonLalabadi, Maryam AziziYoon, GwanhoLalam, NageswaraYoon, Jung HoLambert, PierreYoon, JunsikLang, JochenYoon, MinyoungLantz, Mark A.Yoshino, KenjiLaohakunakorn, NadanaiYou, Jae BemLara Molina, Fabian AndresYou, ShuzhenLau, Gih KeongYoun, HongseokLäubli, Nino F.Younes, HammadLauzurica, SaraYoung, StephenLazarus, NathanYounis, YaminLe Floch, PaulYousefi, MahdiLe Gac, SéverineYu, Chin-PingLe Pioufle, BrunoYu, FabiaoLebedev, Alexander AlexandrovichYu, HongyuLecler, SylvainYu, JiaquanLee, Amos ChungwonYu, NanLee, AnnYu, Ruei-SungLee, Byeong HaYu, ShuyouLee, Byoung-SunYu, TianyuLee, Byung ChulYuan, ChaoLee, Chang-ChunYuan, FengLee, Chang-SeukYun, JinHyeonLee, ChengkuoZagni, NicolòLee, DoojinZahn, JeffreyLee, ElaineŻak, KrzysztofLee, HyochangZakaria, Mohamed B.Lee, Hyun HoZakharov, Alex V.Lee, HyunkeunZamboni, RiccardoLee, Hyun-TaekZamfir, Lucian-GabrielLee, InheeZamorano-Leon, José JavierLee, JaewooZand, RamtinLee, Jeong HoonZandrini, TommasoLee, Jeong-SooZarastvand, MohamadrezaLee, JiseokZareinia, KouroshLee, JongsuZeck, GüntherLee, JongwanZeiler, MatthiasLee, Jue-HyunZekry, AbdelhalimLee, KyueuiZeljkovic, MilanLee, Man-SeopZeng, HaoLee, Moon GuZglobicka, IzabelaLee, SanghoonZha, JunLee, Seok JaeZhang, ChaominLee, Soo-HongZhang, GongLee, Sung SikZhang, GuangpingLee, Tae YoonZhang, HanLee, Yan Jin LionelZhang, HongbinLefik, MarekZhang, JianLei, ShutingZhang, JiaweiLei, SidongZhang, JieLei, ZuhaiZhang, JihuaLeïchlé, ThierryZhang, NaiyinLeissner, TillZhang, NanLemaitre, GerardZhang, PengLemma, EnricoZhang, QiLengvarský, PavolZhang, QichunLeonardi, Antonio AlessioZhang, ShaojianLepore, MariaZhang, XuyangLesher-Pérez, Sasha CaiZhao, ChuanzhenLewinska, GabrielaZhao, HaitaoLewis, OwenZhao, HongLi, GaomingZhao, JingzhouLi, GuonengZhao, Quan-QingLi, HoupuZhao, SipeiLi, HuaizhongZheng, ErjinLi, IsaacZhou, GuodongLi, JiaZhou, JianLi, Ji-HongZhou, LangLi, Jin-FuZhou, SusanLi, KezhengZhu, ChengLi, LanZhu, HongyangLi, LeZielińska-Raczyńska, SylwiaLi, LiuanZizzari, AlessandraLi, LongZolfagharian, AliLi, Ming


